# A Technologist’s Vigilance: Identifying and Correcting a Cotton Ball-Induced MRI Artefact to Prevent Misdiagnosis in Pediatric Patients

**DOI:** 10.7759/cureus.81252

**Published:** 2025-03-26

**Authors:** Longping Liu, Nan Zhou, Xiaoli Zheng, Weiguo Cao

**Affiliations:** 1 Department of Radiology, Shenzhen Children’s Hospital, Shenzhen, CHN

**Keywords:** cotton ball, misdiagnosis, mri artifact, pediatric imaging, quality control

## Abstract

Wrap-around artefacts in magnetic resonance imaging (MRI) are common, typically caused by anatomical structures outside the Field of View (FOV) overlapping into the imaging area. This paper reports a rare source of wrap-around artifact, a wet cotton ball, whose image was inadvertently included in cranial imaging, potentially leading to misdiagnosis as pathological conditions such as otomastoiditis, postoperative changes, or intracranial hemorrhage. Hence, there is a need to enhance MRI technologists' awareness of such artifacts. We analyzed four consecutive cases of cranial MRI scans with similar artifacts, all located on the right side of the brain but in different regions. On axial T2-weighted images (T2WI) and T2 fluid-attenuated inversion recovery (T2FLAIR) sequences, the artifacts appeared as oval-shaped, relatively well-defined heterogeneous high signals. On axial T1-weighted images (T1WI), signal intensity and border clarity varied, with artifact locations slightly more lateral compared to T2WI and T2FLAIR. The artifacts in the first three cases were not identified by the MRI technologists. However, in the fourth case, a patient with head trauma, the MRI technologist noticed inconsistencies in the artifact characteristics across different sequences, as well as similarities to a previous case of cranial trauma, which raised suspicion of an artifact. Systematic image analysis and equipment inspection subsequently revealed a wet cotton ball attached to the outer surface of the head-and-neck coil, inadvertently left there by a radiology nurse during preparation for a previous case. Upon removal of the cotton ball and rescanning the sequences with artifacts in cases 3 and 4, the artifacts disappeared, confirming the wet cotton ball as the source. Additionally, upon review, similar artifacts were found in the first two cases but overlooked due to various reasons. The aim of this study is to improve MRI technologists' recognition of artifacts caused by non-metallic foreign objects, avoiding misdiagnosis, and to prompt us to refine our examination protocols and enhance radiology nurses' awareness of MRI safety.

## Introduction

Magnetic resonance imaging (MRI) is a non-invasive medical imaging technique widely used in clinical diagnostics due to its exceptional soft tissue contrast. However, MRI image quality can be compromised by artifacts, which not only affect the aesthetics of the images but also interfere with the observation and diagnosis of lesions, potentially leading to misdiagnosis [1]. Therefore, technologists and nurses must have a thorough understanding of MRI contraindications and assess the compatibility of medical devices to ensure the safety of pediatric patients undergoing examination.

MRI technologists play a crucial role in ensuring both image quality and patient safety. Their responsibilities include not only executing standardized scanning protocols but also conducting real-time artifact detection and troubleshooting. Despite highly standardized workflows, subtle errors, such as improper handling of non-metallic consumables, can lead to systematic artifacts. These artifacts may be misdiagnosed as pathological changes, thereby triggering diagnostic errors.

Artifacts, which refer to false anatomical structures or pathologies present in the image but not in the body, can degrade MRI image quality. In MRI, artifacts can be technical in origin, related to the patient's state (e.g., patient non-compliance), or caused by foreign objects near or within the patient's body during scanning. These factors can introduce false positives in the image. One type of artifact that can affect MRI image quality is the wrap-around artifact. Wrap-around artifacts occur when the size of the anatomical structure being examined exceeds the Field of View (FOV), causing the image of the portion outside the FOV to shift or wrap around onto the next image, primarily in the phase-encoding direction [2]. Mild wrap-around artifacts may affect image aesthetics, while severe artifacts can interfere with the observation and diagnosis of lesions.

This paper presents a new case where a saline-soaked cotton ball was inadvertently placed on the head-and-neck coil by a radiology nurse, resulting in reproducible wrap-around artifacts in four consecutive head MRI examinations. This case underscores the importance of MRI technologists' ability to identify and address artifacts caused by non-metallic foreign objects to avoid misdiagnosis and highlights the need for continuous improvement in examination protocols and enhancing the overall awareness of MRI safety within the radiology team.

## Case presentation

During a lumbosacral MRI examination for a three-year-old patient with suspected tethered cord syndrome, a radiology nurse prepared a saline-moistened cotton ball to mark the sacral dimple (a depression in the lower back, serving as a landmark). Upon realizing the patient lacked a visible dimple, the nurse inadvertently adhered the cotton ball to the right lateral surface of a 20-channel head-neck coil (MRI System: Magnetom Prisma, Siemens Healthcare, Erlangen, Germany). Unfortunately, this oversight went unnoticed, leading to the involvement of four unrelated pediatric patients during their subsequent cranial MRI examinations. 

**Figure 1:**
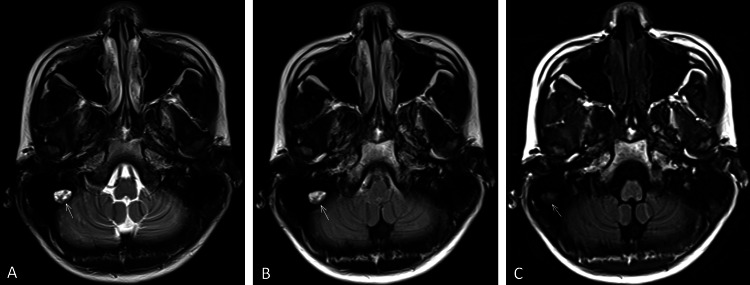
Characterization of the artifact in Case 1 (A) Axial T2 imaging and (B) axial T2FLAIR demonstrate an oval-shaped, well-circumscribed hyperintense artifact (arrows) in the right mastoid region. (C) On Axial T1 imaging, the artifact appears isointense is positioned slightly more lateral compared to its localization on T2 and T2FLAIR sequences (arrow). FLAIR: Fluid-attenuated inversion recovery.

Case 1: A 13-year-old male patient with a clinical diagnosis of attention deficit hyperactivity disorder (ADHD) underwent cranial MRI to rule out any potential brain lesions. Imaging revealed an oval hyperintense lesion (11×8mm) in the right mastoid region on T2WI (TR/TE=2960/106ms) and T2-FLAIR (TR/TE=9000/132ms) sequences. The artifact demonstrated isointensity on T1WI (TR/TE=1800/43ms) and was laterally displaced compared to its position on T2 and T2-FLAIR sequences (Figure 1). The MRI technologist, focusing solely on the details within the brain tissue, overlooked the artifact outside the brain tissue, thereby neglecting its presence.

**Figure 2:**
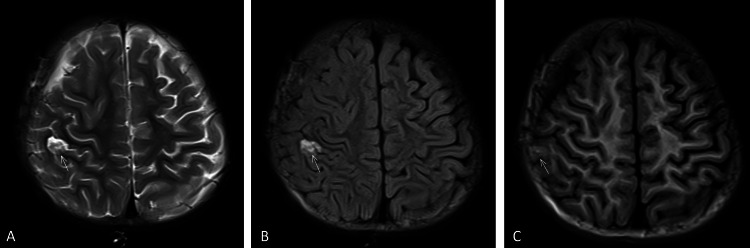
Characterization of the artifact in Case 2 (A) Axial T2 and (B) axial T2FLAIR reveal an oval-shaped uneven hyperintense artifact in the right parietal lobe (arrows). The anterior margin is relatively well-demarcated, while the posterior margin is ill-defined. (C) On Axial T1, a faint, ill-defined patchy hyperintensity (arrow) is observed adjacent to the inner table of the skull, with a lateral displacement compared to its location on T2 and T2FLAIR sequences. FLAIR: Fluid-attenuated inversion recovery.

Case 2: An 18-month-old male patient with a history of right temporal lobe tumor resection underwent a follow-up MRI for postoperative surveillance. Imaging revealed an oval hyperintense lesion (11×8mm) in the right parietal lobe. The lesion exhibited distinct signal characteristics: marked hyperintensity on T2 and T2-FLAIR sequences, mild hyperintensity with ill-defined margins on T1WI, and a more lateral location compared to its appearance on T2WI and T2-FLAIR (Figure 2). Given the extensive surgical modification, the MRI technologist focused primarily on anatomical changes in the surgical region, such as brain tissue displacement and alterations in the cerebrospinal fluid spaces. However, during this process, artifacts generated (such as those caused by metallic implants or signal abnormalities due to postoperative tissue edema) were mistakenly regarded as normal components of the postoperative changes, thereby neglecting the presence of the artifacts.

**Figure 3:**
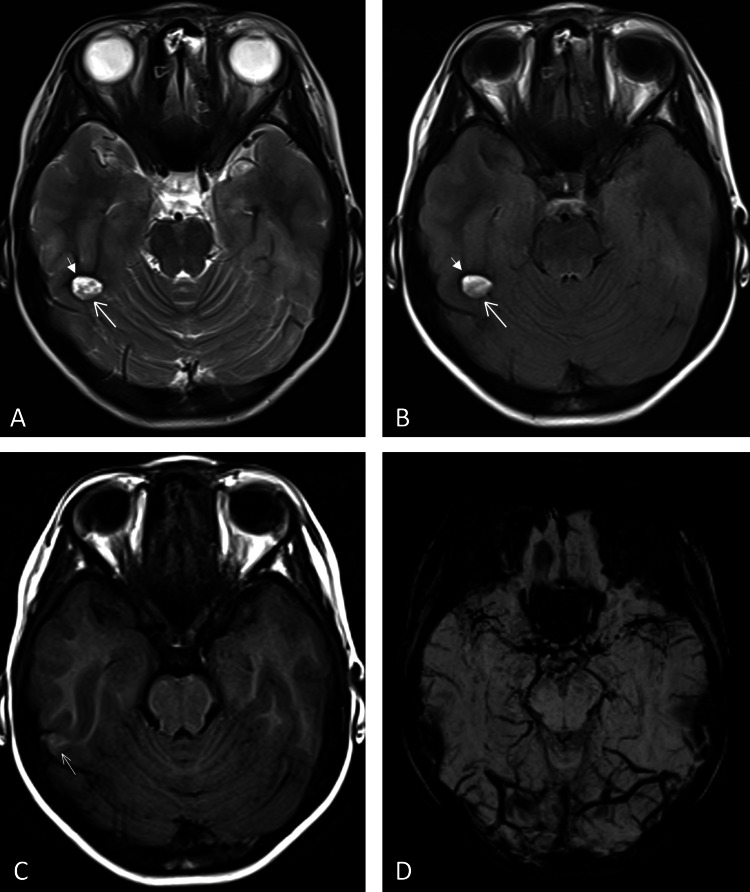
Characterization of the artifact in Case 3 (A) Axial T2 and (B) axial T2FLAIR sequences demonstrate an oval-shaped heterogeneous hyperintense lesion in the right temporal lobe with well-defined margins(arrows). A hypointense rim (short arrows) is visible along the anterior margin, while the posterior margin is absent. On (C) axial T1, a small patchy moderately hyperintense signal is observed with ill-defined margins, and it is slightly more lateral compared to its location on T2 and T2FLAIR sequences(arrow). On (D) SWI sequences, no abnormal signal is detected at this anatomical location. SWI: Susceptibility-weighted imaging, FLAIR: Fluid-attenuated inversion recovery.

Case 3: An 11-year-old female patient underwent post-traumatic evaluation after a fall. Imaging revealed an oval hyperintensity (11×8mm) in the right temporal lobe with a hypointense rim on T2WI and T2FLAIR sequences. The lesion appeared slightly hyperintense on T1WI, though the signal was not very clear. No abnormalities were detected on susceptibility-weighted imaging (SWI, TR/TE=27/20ms) (Figure 3). During scanning, although abnormal signals were observed and discrepancies among different sequence images were noted, the scanner's ability to recognize artifacts was limited. There were apparent differences in the interpretation of pathology between T1, T2, and T2FLAIR sequences versus SWI images. However, due to a lack of sufficient diagnostic knowledge and experience, the scanner failed to promptly identify these discrepancies as potentially originating from artifact interference. Instead, the scanner erroneously attributed them to hemorrhagic lesions.

**Figure 4:**
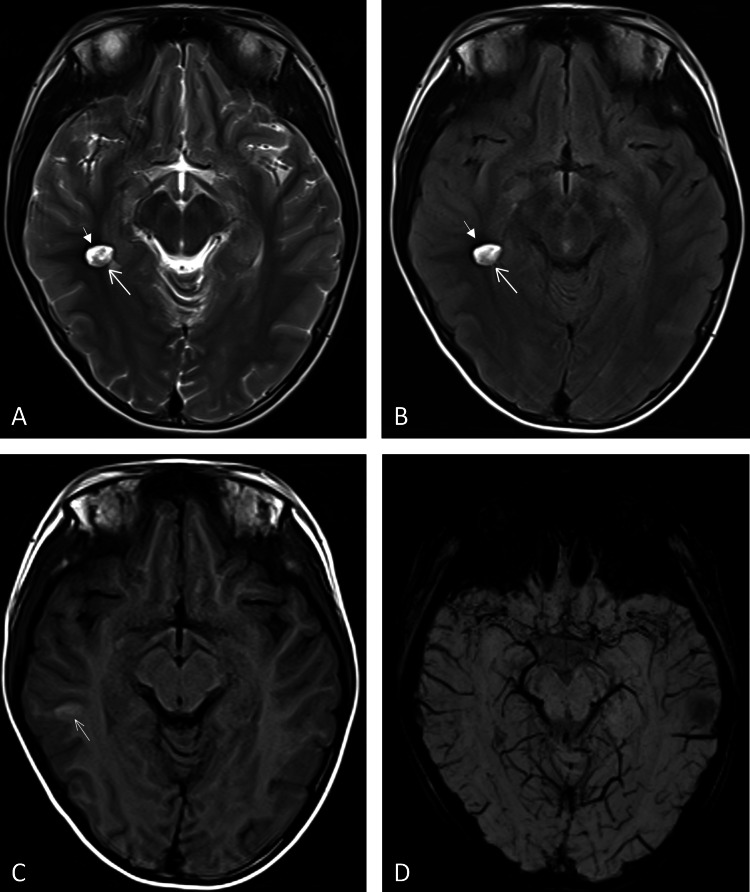
Imaging findings of Case 4 Artifact mirroring Case 3 (A-D, arrows).

Case 4: A 10-year-old female patient presented with head trauma. Imaging revealed an artifact identical to that described in Case 3 (Figure 4). The MRI technologist noted that the lesion’s fixed size, shape, and location were consistent with those observed in Case 3, despite the differing clinical contexts. This observation alerted the technologist to the possibility of an artifact rather than a true pathological finding.

**Figure 5:**
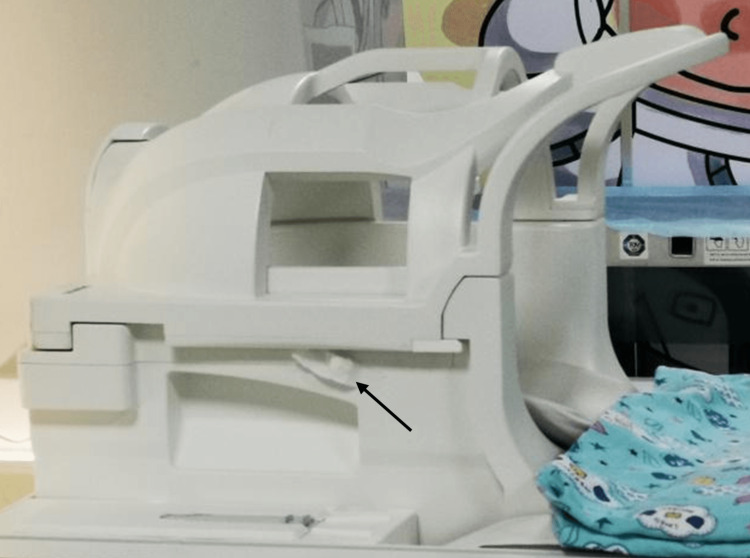
The position of the wet cotton ball on the coil. Saline-moistened cotton ball adhered to the right lateral surface of the head-neck coil (black arrow).

**Figure 6:**
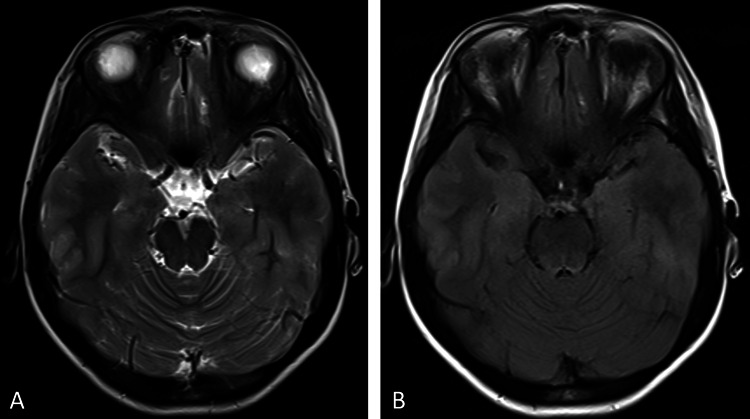
Re-scan images of Case 3 Artifact resolution in (A) T2 and (B) T2FLAIR sequences. T1 was not re-scanned. FLAIR: Fluid-attenuated inversion recovery.

**Figure 7:**
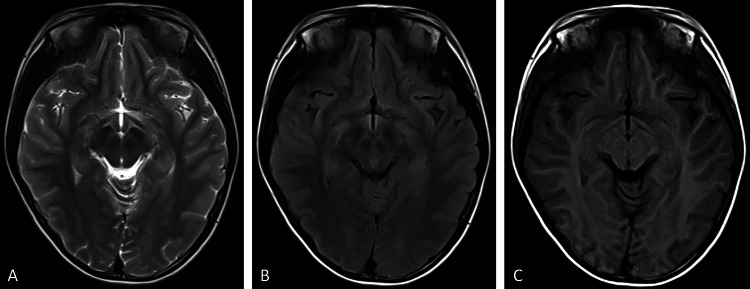
Re-scan imagings of Case 4 Artifact resolution in (A) T2, (B) T2FLAIR and, (C) T1 sequences. FLAIR: Fluid-attenuated inversion recovery.

A systematic review of the cases revealed identical artifact morphology (11×8mm oval) and fixed laterality across all four cases, highlighting the consistency of the artifact across different patients and scans. Upon inspection, a retained saline-moistened cotton ball was discovered adhered to the right lateral surface of the head-neck coil (Figure 5). After removing the cotton ball, re-scans of Cases 3 and 4 were performed. Repeat T2, T1, and T2-FLAIR sequences confirmed the resolution of the artifact (Figures 6, 7), validating the initial suspicion and preventing further misdiagnoses. Cases 1 and 2 were retrospectively identified. Case 1 was not re-scanned because the artifact was located outside the brain tissue and did not affect the evaluation of the patient's ADHD. Case 2, an infant, was not re-scanned due to the need for additional sedation, which was deemed unnecessary. The radiologists were informed to correct the misdiagnoses, thereby avoiding unnecessary interventions for these patients.

## Discussion

Wrap-around artifacts, common in MRI, particularly in the phase encoding direction, stem from insufficient spatial encoding during Fourier transformation, causing signal misregistration. In our cases, the artifacts were attributed to two factors: (1) the use of a left-right phase-encoding direction and (2) the absence of phase oversampling. The left-right phase-encoding direction caused signals from the wet cotton ball (positioned outside the FOV) to fold into the temporal/parietal regions. Additionally, the lack of phase oversampling further exacerbated the aliasing by limiting the spatial resolution in the phase-encoding axis [3].

The artifact location was modulated by the FOV size. Smaller FOVs resulted in central artifact positioning due to narrower frequency intervals, whereas larger FOVs shifted artifacts toward the periphery [4]. This aligns with the Nyquist theorem, where smaller FOVs increase the risk of high-frequency signal aliasing [5]. In this case series, the T2 and T2FLAIR sequences used a smaller left-right FOV (230×84.4% mm), while the T1 sequence used a larger left-right FOV (230×93.8% mm). Due to the larger FOV in T1WI, signals outside the imaging FOV had to cross a greater spatial distance when wrapping around, resulting in artifact positions that were relatively more lateral.

The differences in artifact location between infants and older children correlated with anatomic variations. In older children, longer necks positioned the head deeper within the coil, projecting artifacts to the temporal/mastoid regions adjacent to the coil’s lateral surface (cases 1, 3, and 4). Conversely, infants’ shorter necks led to superficial head placement, redirecting artifacts to the parietal lobe (case 2). This spatial pattern follows the "closest-adjacent principle," where out-of-FOV signals fold into anatomically proximate regions [4].

The wet cotton ball exhibited distinct signals on different sequences. On T2WI, the cotton ball appeared hyperintense due to its water content, which has a long T2 value. However, the water in the cotton ball may combine with the cotton fibers to form bound water, which has T2 values longer than brain tissue but shorter than free cerebrospinal fluid (CSF). This results in a T2 signal intensity that is not as high as that of cerebrospinal fluid CSF [6]. On T1WI, the cotton ball appeared isointense or slightly hyperintense, as the combination of water with cotton fibers led to a relatively shorter T1 value [6]. On T2FLAIR sequences, the signal from the cotton ball remained hyperintense despite the inversion recovery technique used to suppress the high signal of CSF. This may be due to the T2 value of bound water (~100-500ms) falling below the FLAIR suppression threshold (typically >2000ms), resulting in residual hyperintensity [7]. 

A hypointense rim was observed along the anterior margin of the artifact in Cases 3 and 4 (absent posteriorly), which may arise from multiple mechanisms. Magnetic field inhomogeneity could be a contributing factor, as the anterior margin is farther from the coil, where the magnetic field may be more inhomogeneous. This leads to a shortened T2 relaxation time of the signal, presenting as a hypointense rim. Additionally, the partial volume effect may play a role, as the anterior margin is closer to the petrous part of the temporal bone, which contains more air and bone. This results in a reduction of signal intensity due to the partial volume effect. Further investigation may be required to elucidate the specific mechanisms [8,9].

Susceptibility-weighted imaging (SWI) did not display the wrap-around artifacts due to its inherent technical properties. Mechanistically, SWI enhances image contrast by exploiting the susceptibility differences between tissues, primarily detecting structures such as veins, microbleeds, and calcifications rather than T1/T2 relaxation changes caused by bound water [10]. Additionally, SWI often uses three-dimensional (3D) gradient echo sequences with smaller voxels and thinner slice thickness, combined with a larger FOV design. These features significantly reduce the risk of wrap-around artifacts. Furthermore, SWI enhances contrast through high-pass filtering and phase masking techniques, which may effectively filter out the low-frequency signals caused by cotton ball artifacts, making them not obvious on SWI images [11].

To reduce similar artifacts, we propose the following measures. First, we recommend pre-scan standardization by conducting pre-scan preparations in a designated area outside the magnet room to remove non-metallic foreign objects such as cotton balls, electrodes, and diapers. Second, we suggest establishing a multidisciplinary collaboration system. When MRI technologists encounter unexplainable abnormal signals, they should be able to consult with senior technologists or radiologists in real time to clarify ambiguous signals. Finally, we propose providing quarterly MRI safety training for radiology technologists and nurses to enhance their awareness of MRI safety, particularly the impact of non-metallic foreign objects on MRI imaging. In summary, our findings underscore the critical need for vigilance in identifying and addressing artifacts in MRI imaging, particularly those caused by non-metallic foreign objects.

## Conclusions

This study analyzed four pediatric cranial MRI cases where artifacts caused by a saline-moistened cotton ball adhered to the MRI coil led to misdiagnosis. The key findings include the characteristic appearance of the cotton ball artifact on different MRI sequences, the reasons for its formation, and the reasons for the technologist's oversight. Through systematic case review, coil inspection, and re-scanning after removing the cotton ball, the artifact was confirmed to originate from the cotton ball. These findings highlight the importance of increasing awareness of artifacts caused by non-metallic foreign objects, optimizing pre-scan preparation protocols, and enhancing training for technologists and nurses to prevent misdiagnoses due to such artifacts. Ultimately, this study provides valuable insights for clinical practice and future research directions, contributing to the accuracy and safety of MRI examinations.
